# The A487 residue in the E protein of duck Tembusu virus significantly enhances viral replication and increases its neurovirulence in Kunming mice

**DOI:** 10.1128/jvi.00308-25

**Published:** 2025-05-22

**Authors:** Yu He, Jiaqi Guo, Xiaoli Wang, Zhen Wu, Tao Wang, Mingshu Wang, Renyong Jia, Dekang Zhu, Mafeng Liu, Xinxin Zhao, Qiao Yang, Ying Wu, Shaqiu Zhang, Juan Huang, Xumin Ou, Di Sun, Anchun Cheng, Shun Chen

**Affiliations:** 1 Institute of Veterinary Medicine and Immunology, Sichuan Agricultural University 506176, Chengdu, Sichuan, China; 2 Research Center of Avian Disease, College of Veterinary Medicine, Sichuan Agricultural University 506176, Chengdu, Sichuan, China; 3 Key Laboratory of Animal Disease and Human Health of Sichuan Province, Sichuan Agricultural University 506176, Chengdu, Sichuan, China; 4 Key Laboratory of Agricultural Bioinformatics, Ministry of Education, Sichuan Agricultural University 506176, Chengdu, Sichuan, China; 5 Engineering Research Center of Southwest Animal Disease Prevention and Control Technology for Ministry of Education, Sichuan Agricultural University 506176, Chengdu, Sichuan, China; University of Michigan Medical School, Ann Arbor, Michigan, USA

**Keywords:** Tembusu virus, flavivirus, neurovirulence, E protein, assembly

## Abstract

Tembusu virus (TMUV), an emerging avian orthoflavivirus, causes severe egg-drop syndrome and encephalitis in ducks. Although ducks are the natural host, mice serve as a valuable model for studying neuropathogenesis, as TMUV-infected mice recapitulate key neurological symptoms observed in ducks, such as paralysis and encephalitis. In the previous study, we observed that the TMUV strain CQW1 exhibited unexpectedly low neurovirulence in mice compared with earlier strains, highlighting potential genetic determinants of pathogenicity that may influence viral evolution and disease outcomes in natural hosts. In this study, we investigated the murine neurovirulence of TMUV strains from two major phylogenetic clusters (2.1 and 2.2). The Cluster 2.2 strain CHN-YC demonstrated markedly higher neurovirulence in Kunming mice than Cluster 2.1 strains (CQW1 and SCS01), with robust viral replication in the brain, pronounced histopathological damage, and elevated proinflammatory cytokine levels. Comparative genomic analysis identified seven amino acid substitutions in the E-NS1 region, with variations unique to Cluster 2.1 strains or specific to CQW1. By introducing these substitutions into CQW1 via reverse genetics, we restored high murine neurovirulence and identified the E protein substitution V487A as critical for this phenotype. Mechanistically, E-V487A enhances viral assembly, which boosts replication efficiency *in vitro* and *in vivo*. This substitution is located in the E protein transmembrane domain, a region implicated in flavivirus particle formation. Our data revealed that a naturally occurring amino acid substitution located in the transmembrane domain of the Tembusu virus E protein is responsible for its high neurovirulence in mice.

**IMPORTANCE:**

Tembusu virus is a mosquito-borne avian orthoflavivirus, exhibiting airborne transmission. Although it primarily affects domestic fowl, TMUV demonstrates high neurovirulence in mice during laboratory studies and has been reported to spill over into humans. Recent years have seen increased genetic diversity and an expanded host range of the virus. Strains belonging to phylogenetic cluster 3 can cause severe neurological symptoms and death in mice via intranasal infection, further highlighting its risk of potential transmission to mammals. Understanding their pathogenicity and the underlying molecular basis is crucial for assessing and preventing health risks to mammals. We identified a single amino acid substitution in the TMUV E protein that critically enhances viral replication and neurovirulence in mice. The data provide insights into the molecular mechanisms of Tembusu virus pathogenesis in mammals and underscore the impact of specific genetic mutations on the viral phenotype.

## INTRODUCTION

The genus *Orthoflavivirus* includes several significant viral pathogens that pose a considerable threat to global public health, such as Zika virus (ZIKV), dengue virus (DENV), Japanese encephalitis virus (JEV), and yellow fever virus (YFV). Most known flaviviruses are zoonotic pathogens transmitted by mosquitoes or ticks and are responsible for diverse diseases in humans and animals. Among these, Tembusu virus (TMUV) is an emerging mosquito-borne flavivirus that causes severe neurological and reproductive diseases in birds.

Like other flaviviruses, TMUV is a single-stranded positive-sense RNA virus with an envelope. Its viral genomic RNA is approximately 10,990 nt in length and contains a single open reading frame encoding a 3,425-aa polyprotein. Following cleavage by viral NS2B/3 protease and host signalase, this polyprotein generates three structural proteins (C, prM, and E) and seven nonstructural proteins (NS1, NS2A/2B, NS3, NS4A/4B, and NS5). The structural proteins, along with viral RNA, form the virion. The E protein is the major envelope protein, which has three domains (Domains I–III) at the N-terminus and two transmembrane regions at the C-terminus, connected by a stem region. The nonstructural proteins play crucial roles in various key processes of the viral life cycle, including viral RNA replication, assembly, and evasion of host immunity (1, 2).

TMUV was first identified in *Culex tritaeniorhynchus* mosquitoes in Malaysia in 1955 and has maintained its transmission cycles within mosquito populations (3). In 2010, a severe duck egg-drop syndrome epidemic caused by TMUV suddenly emerged and quickly spread throughout mainland China, resulting in enormous economic losses to poultry farming (4, 5). Subsequent studies indicate that TMUV has an extensive natural host range, including chickens, sparrows, geese, and multiple mosquito species (e.g., *Culex quinquefasciatus* and *Culex tritaeniorhynchus*) (6). Laboratory studies using needle inoculation indicated that TMUV is highly virulent to BALB/c mice and Kunming mice by intracerebral inoculation (7–10). *In vitro* studies have also shown that TMUV can replicate well in a wide spectrum of mammalian, avian, and mosquito cells (6). Additionally, high rates of seropositivity and viral RNA positivity for TMUV have been detected in duck farm workers in China (11). Residents around farming areas who had or had no contact with ducks also displayed high neutralizing antibody titers to TMUV in Thailand (12). These data suggest the possibility of TMUV as a potential zoonotic pathogen.

TMUV is thought to originate in Southeast Asia, as the prototypical TMUV MM_1775 strain was isolated from *C. tritaeniorhynchus* in 1955 (13). A retrospective study also indicated the presence of TMUV in Thai ducks since 2007, prior to the outbreak of duck TMUV in mainland China (14). Phylogenetic analysis highlights the high genetic diversity of TMUV in Asia. Cluster 1 TMUV has only been isolated from the Southeast Asia region and showed less pathogenicity than cluster 2 TMUV (15). Currently, circulating duck TMUVs belong to Cluster 2, subdivided into Clusters 2.1 and 2.2. Laboratory infections indicate that viruses from both subclusters showed a high pathogenicity to ducks, causing similar disease symptoms, such as loss of appetite, conjunctivitis, and severe neurological signs, including ataxia, reluctance to walk, and paralysis (16, 17). The recent epidemic in chickens and geese was caused by Cluster 3, which mainly consists of mosquito TMUVs (18–22). This cluster TMUV caused symptoms in chickens similar to those observed in ducks but is more virulent to chickens than ducks. The expanding natural host range of TMUV highlights the continuous evolution and variation of TMUV, warranting attention.

Mice are widely used as an animal model for studying encephalitis-related orthoflavivirus infections. Laboratory studies using needle inoculation have demonstrated that TMUV-infected (Kunming) mice develop neurological symptoms similar to those observed in ducks (conjunctivitis, ataxia, reluctance to walk, and paralysis), making them a suitable model for investigating the neurovirulence of TMUV (8, 23). In this study, we found that the TMUV strain within Cluster 2.1 displayed attenuated neurovirulence in Kunming mice compared to the Cluster 2.2 strain. Using reverse genetics technology, we analyzed the viral determinants in detail and confirmed that a single amino acid substitution located in the transmembrane domain of the E protein is responsible for the altered neurovirulence.

## MATERIALS AND METHODS

### Cells and viruses

Baby hamster kidney cells (BHK-21) were maintained in Modified Eagle’s medium (DMEM) (Gibco, Shanghai, China) with 10% fetal bovine serum (FBS) (Gibco, New York, USA) and incubated at 37°C with 5% CO_2_. Duck embryo fibroblast (DEF) cells were obtained from 9-day-old duck embryos using a general procedure, maintained in DMEM (Gibco, Shanghai, China) with 10% newborn calf serum (Gibco, New York, USA), and incubated at 37°C with 5% CO_2_.

The duck TMUV strain CQW1 (GenBank: KM233707.1) was rescued using reverse genetics technology (24). The recent TMUV strain CHN-YC (GenBank: MN966680.1) was isolated in 2019, and strain MC (GenBank: KX452096.1) was isolated in 2014 (both strains are gifts from Professor Rui Luo, Huazhong Agricultural University). Another recent TMUV strain, SCS01 (GenBank: MW143073.1), was also isolated in 2019 by our lab. All of these viruses have never been passaged and adapted in mouse brains, and virus stocks were prepared in BHK-21 cells.

### Generation and confirmation of recombinant viruses

Recombinant TMUV with seven substitutions (E-N277S/V487A, and NS1-K48E/T112M/ A274V/I318T/I338T) was generated using the infectious subgenomic amplicon method (25). Briefly, DNA fragment P1, covering the 1^st^ to 4,018^th^ nucleotides and containing the seven mutations (with a CMV promoter at its 5′-terminus), was engineered by overlapping PCR. Fragment P2, covering the 3,934^th^ to the end nucleotides with an SV40 poly(A) tail, was amplified by PCR using the Phanta HS Super-Fidelity DNA Polymerase kit (Vazyme, Nanjing, China). TMUV full-length cDNA infectious clone pACNR-CQW1-Intron (24), or pACYC FL-CQW1 plasmids (26) served as templates. When these fragments were transfected into a single cell, they spontaneously recombine and synthesize a DNA copy of the entire viral genome. For the seven single-mutation viruses, each mutation was engineered by overlapping PCR to generate a full-length infectious cDNA clone based on the pACNR-CQW1-Intron plasmid. All of the related primers are presented in Table 1.

**TABLE 1:** Primers used for generation of recombinant viruses

No.	Primer name	Sequence (5'−3')	Note
1	E-N277S-F	GTGAAGTACTCTGGAAGCAAATTGGAAATG	Primers for the seven mutations
	E-N277S-R	CTTCCAGAGTACTTCACTGGAATAGCTCCC	
2	E-V487A-F	TTTCTATGACCTTTCTAGCCGTAGGAGGAAT	
	E-V487A-R	GCTAGAAAGGTCATAGAAATGGATCTGTCCC	
3	NS1-K48E-F	GCCAAAGTCGTGGCAGAAGCTCATGAG	
	NS1-K48E-R	CTGCCACGACTTTGGCAAGTCTCCTTG	
4	NS1-T112M-F	ACGTTGATGGAGAGCTCATGTACGGATGG	
	NS1-T112M-R	ATGAGCTCTCCATCAACGTTCGGCAG	
5	NS1-A274V-F	GGGATGAGAAAGAGATTGTAATAGACTTCG	
	NS1-A274V-R	TACAATCTCTTTCTCATCCCACGGTCC	
6	NS1-I318T-F	GTAGGTCTTGCACCACCCCACCACTG	
	NS1-I318T-R	GTGGTGCAAGACCTACAACACCAATCTGTTA	
7	NS1-I338T-F	ATGGAAATTCGGCCAACTGTTCACGGA	
	NS1-I338T-R	GTTGGCCGAATTTCCATCCCATACCA	
8	PACNR-P1-F	gtatcatacacatacgACGCGTTGGAGTTCCGCGTTACATAACTT	Primers for SAP
	PACNR-P1-R	TCTATCCCCACTATTCTGAGTCCTG	
9	PACNR-P2-F	GAGCCGTGTTTGAAGGGACGGTT	
	PACNR-P2-R	tcaacgggaaacgtcttgTCGCGATAAGATACATTGATGAGTT	

For the recovery of recombinant viruses, BHK-21 cells were seeded in 12-well plates and cultured to 70%–90% confluence, then transfected with 1 µg of an equimolar mix of the two described DNA fragments per well using Lipofectamine 3000 (Thermo Fisher Scientific, Shanghai, China) according to the manufacturer’s instructions. The cells were continuously cultured at 37°C with 5% CO_2_. When a 70% cytopathic effect (CPE) appeared, the supernatant was harvested, clarified by centrifugation, stored at −80°C, or used for preparing F1 virus stocks then subjected to whole-genome sequencing to validate viral sequence authenticity.

At 3 days post-infection, TMUV production was validated by indirect immunofluorescence assay (IFA) as previously described (26). Briefly, cells growing on glass coverslips were washed twice with PBS, fixed with 4% paraformaldehyde for 1 h, and then permeabilized for 30 min at 4°C with 0.3% Triton in PBS. After 1 h of blocking at 37°C with 5% bovine serum albumin (BSA) in PBS, the cells were incubated with anti-TMUV mouse polyclonal antibody (self-prepared, 1:200 diluted in PBS containing 1% BSA) for 2 h, followed by incubation with goat anti-mouse IgG conjugated with fluorescein 5-isothiocyanate (FITC, Thermo Fisher Scientific, Shanghai, China; catalog #A16067; 1:1000 dilution) for 1 h. Finally, the cells were then stained with 4',6-diamidino-2-phenylindole (DAPI, Coolaber, Beijing, China) in PBS for 10 min. Each step was followed by three 5 min washes with ice-cold phosphate buffered saline with 1‰ Tween-20 (PBST) in an orbital shaker. Fluorescence images were acquired under a fluorescence microscope (Nikon, Tokyo, Japan).

### Virus titration, growth kinetic curve, and plaque assay

Viral titers were determined by the median tissue culture infection dose (TCID_50_) on BHK-21 cells, following procedures as previously reported (26).

For the virus growth curve, BHK-21 cells were pre-cultured in 24-well plates to 80% confluence. The cells were washed twice with PBS and then infected with virus samples at a 200 TCID_50_ (MOI ≈ 0.005). After 1.5 h of incubation at 37°C, the supernatant was removed, the cells were washed with PBS, and then supplemented with DMEM containing 2% FBS and 1% penicillin/streptomycin. The plates were incubated at 37°C with 5% CO_2_. Cell supernatants were sampled every 12 h at indicated time points and stored at −80°C until used for virus titration.

The plaque assay was performed as described previously (27). Briefly, 300 µL of serially diluted virus samples (in DMEM) were distributed into wells of a 12-well plate seeded with nearly confluent BHK-21 cells. After 1.5 h of attachment at 37°C, the inocula were removed and replaced with 1 mL of 1% methylcellulose overlay containing 2% FBS and 1% penicillin/streptomycin. At 5 days post-infection, the overlay was carefully removed, and the cells were washed twice with PBS, fixed with 4% formaldehyde at room temperature for 20 min, and then stained with 1% crystal violet for 1 min. The cells were washed carefully with running water, and visible plaques were observed. Cell plates are photographed under white lighting. To assess the plaque size of the virus, the captured plaque images were processed using Image J software (https://imagej.net/ij/) and created a binary image (plaques = black and background = white). Then, the data for the area of randomly selected plaques were recorded and exported for analysis using GraphPad Prism software.

### Virulence in duck embryos

Duck embryos were purchased from the Waterfowl Breeding Center of Sichuan Agricultural University. Four groups of 9-day-old embryo eggs (10 per group) were injected with 100 µL TMUV (10^3^ TCID_50_), and the mock group was injected with the same dose of DMEM. Then, the eggs were incubated in an egg incubator at 37°C. The eggs were checked daily, and the survival time was recorded.

### Mouse study design and TMUV infections *in vivo*

Kunming Mice were purchased from Chengdu DOSSY Experimental Animals CO., LTD (Chengdu, China). Three-week-old female mice were randomly divided into groups of 8 or 10 mice each. Each mouse was infected with 30 µL of TMUV (10^3.98^ or 10^4.98^ TCID_50_) via intracerebral (i.c.) injection or 200 µL via intraperitoneal (i.p.) injection. For the mock control, mice were injected with the same dose of DMEM. Weight changes, clinical signs, and mortalities were monitored and recorded every day. Mice exhibiting body weight loss of more than 20% or complete hind leg paralysis were euthanized. The clinical symptoms of the mice were scored according to severity: (i) low spirits and slow movement, (ii) paralysis of the legs, (iii) inability to open eyes due to discharge, and (iv) death. For each item, a score of 1 corresponded to one mouse showing the corresponding symptom. The total points were calculated as 10% * Item 1 + 30% * Item 2 + 15% * Item 3 + 45% * Item 4.

To detect virus replication *in vivo*, at 3/5 days post-infection, three additional mice in each group were randomly selected for euthanization by humans, and organ samples (i.e., heart, liver, spleen, lung, kidney, brain, and duodenum) were obtained in duplicate; a portion of tissue samples was frozen at −80℃ until used for viral titration or RNA extraction, and the other portion was fixed in 10% neutral buffered formalin for histopathology processing.

### RNA extraction and real-time quantitative reverse transcription-PCR (RT–qPCR)

The isolation of total RNA and RT–qPCR detection were performed as previously reported (27). Total RNA was isolated using RNAiso Plus reagent (Takara, Dalian, China) according to the manufacturer’s instructions. The isolated RNA was then used as a template for synthesizing first-strand cDNA using a HiScript III 1st Strand cDNA Synthesis Kit (Vazyme, Nanjing, China). To measure viral RNA or the transcriptional expression of cytokines, qPCR assays were performed using 2 × Taq SYBR Green qPCR Premix (Innovagene, Changsha, China) with a CFX Connect Real-Time PCR Detect System (Bio–Rad) following the manufacturer’s protocols. All of the related primers for RT-qPCR are presented in Table 2.

**TABLE 2:** Murine probes used for real-time RT-PCR

Gene	Sequence 5'→3'	
GAPDH	F	GATGGACACATTGGGGTT
	R	AAAGCTGTGGCGTGATG
IFN-β	F	TTACACTGCCTTTGCCATCC
	R	ACTGTCTGCTGGTGGAGTTCAT
IFN-α	F	AATGCAACCCTCCTAGACTC
	R	GGCTCTCCAGACTTCTGCTC
IFN-γ	F	ACTGGCAAAAGGATGGTG
	R	GTTGCTGATGGCCTGATT
IL-10	F	GCCCTTTGCTATGGTGTC
	R	TCTCCCTGGTTTCTCTTCC
lL-6	F	GTATGAACAACGATGATGCAC
	R	CTCCAGAAGACCAGAGGAAA
IL-1β	F	AGTTGACGGACCCCAAA
	R	TCTTGTTGATGTGCTGCTG
Mmp3	F	GCTCATCCTACCCATTGC
	R	GCTCCATACCAGCATCATC
TNF-α	F	CGCTGAGGTCAATCTGC
	R	GGCTGGGTAGAGAATGGA

### Histopathology and immunohistochemistry

Histopathology and immunohistochemistry were performed according to a previously described protocol (28). Mouse brain samples were fixed in buffered 10% formalin for 24 h, followed by dehydration in graded alcohol, embedding in paraffin wax, and cutting into 5-μm-thick sections. Some sections were stained with hematoxylin and eosin (H&E) using a conventional protocol. Concurrently, other sections were subjected to immunohistochemical (IHC) staining using a Biotin-Streptavidin HRP Detection System (ZSGB-BIO, Beijing, China) according to the manufacturer’s protocol. Sections were incubated overnight with a mouse polyclonal antibody against TMUV E protein (1:300 dilution) at 4°C. Then, sections were treated with a goat anti-mouse secondary antibody (ZSGB-BIO, Beijing, China) for 30 min at 37°C. Finally, the sections were observed under an optical microscope (Nikon, Tokyo, Japan).

### Packaging assay

Subgenomic replicon assays were performed similarly to a previous report (29). BHK-21 cells, seeded in 48-well plates and reaching 70-90% confluence, were co-transfected with mC-Replicon-NLuc and pCDNA3.1-C_16_prME plasmids at a ratio of 1:3, using TransIntro EL Transfection Reagent (TransGen Biotech, Beijing, China) according to the manufacturer’s instructions. Six hours post-transfection, the cell medium was replaced with fresh maintenance media. Three days post-transfection, the supernatant, containing single-round infectious particles, was collected and used to infect fresh BHK-21 cells in a new 48-well plate. After 1.5 h of incubation, the inoculum was removed, the cells were washed with PBS, and a maintenance medium was added. At 24 h post-infection, the cells were washed once with PBS and lysed with Glo Lysis Buffer (Promega, WI, USA) at room temperature. Nluc activity, used to assess viral packaging efficiency, was detected using the Nano-Glo Luciferase Assay System (Promega) and the GloMax Navigator System (Promega), following the manufacturer’s instructions.

### Quantification and statistical analysis

The data from the analyses of viral titers and the RT–qPCR data are presented as the means ± standard deviations (SDs), using GraphPad Prism 8.0 software; the statistical significance of viral growth curve and weight change was assessed by two-way ANOVA test; the statistical significance of plaque size was assessed by Student’s *t* test or one-way ANOVA test; gene expression and packaging assay were assessed by one-way ANOVA test; and significance was defined by a *P* value < 0.05 (*), <0.01 (**), <0.001 (***), <0.0001 (****). The statistical significance of survival was analyzed using a survival curve and the log-rank (Mantel–Cox) test with GraphPad Prism 8.0 software, and significance was defined by a *P* value < 0.05 (*).

## RESULTS

### Different TMUV strains exhibited varying neurovirulence in Kunming mice

In our previous laboratory studies, we observed that the CQW1 strain exhibited no significant neurovirulence in 6-week-old mice, contrary to findings reported for other early duck TMUV strains (7–9). We hypothesized that different duck TMUV strains might have evolved distinct characteristics in mice. To test this hypothesis, we selected two additional duck TMUV strains for this study and carefully assessed the *in vivo* characteristics in mice.

**Fig 1:**
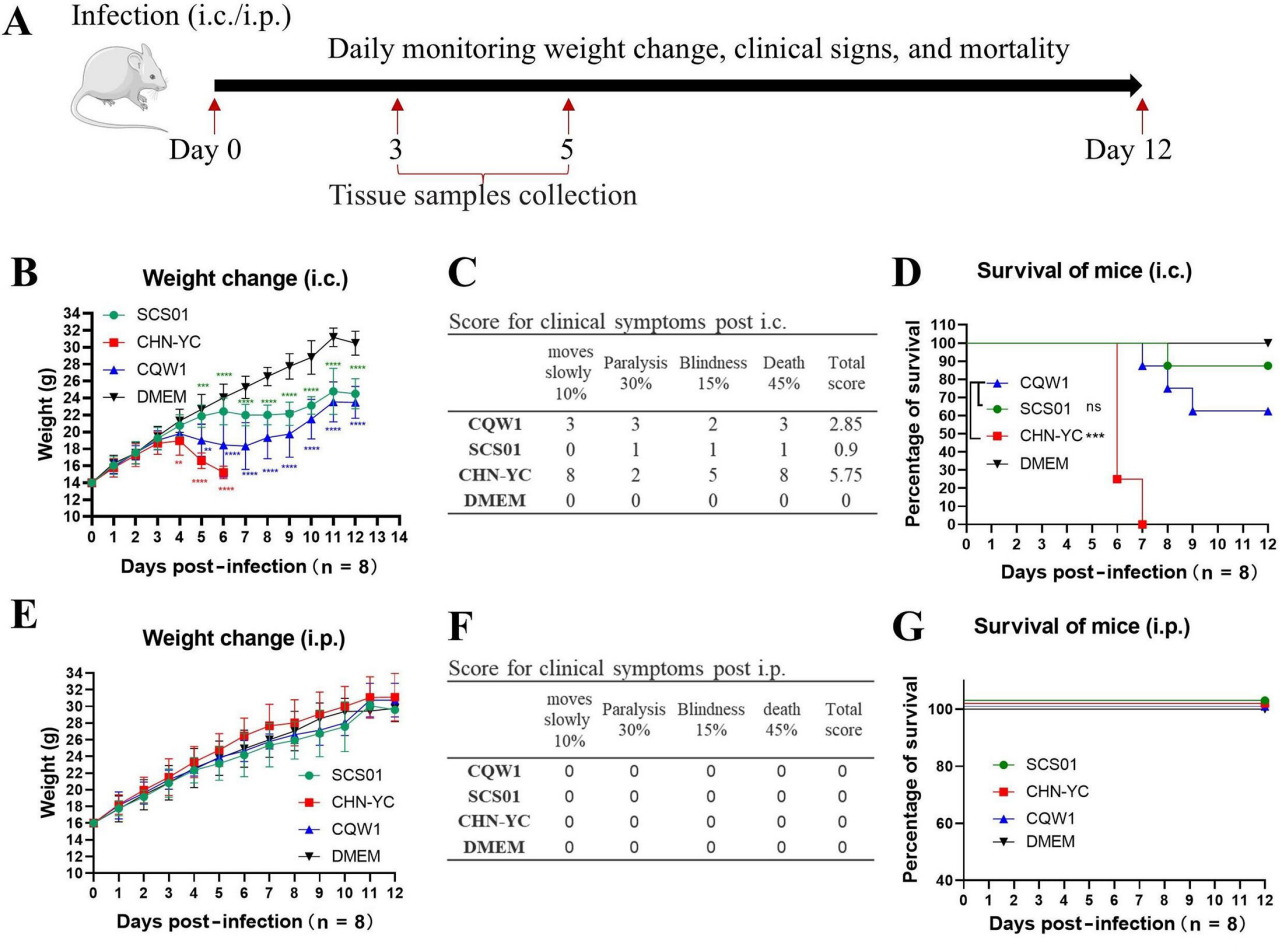
Different TMUV strains exhibited varying neurovirulence in Kunming mice. (**A**) Experimental design for the mice assay. Three-week-old Kunming mice (*n* = 8) were infected with TMUVs via i.c. or i.p. injection. Mouse weight changes, clinical signs, and mortality were monitored daily. Tissue samples were collected at 3 and 5 days post-infection for virus titration, H&E staining, and immunohistochemistry (IHC) analysis. (**B**) Weight changes in mice infected via i.c. injection. The data are presented as the means and SDs; statistical significance was analyzed using two-way ANOVA, defined by *P < 0.05; **P < 0.01; ***P < 0.001, and ns indicates no significance. (**C**) Clinical symptom scores of mice infected with CHN-YC, SCS01, and CQW1 via i.c. injection. Total points = 10% × item 1 + 30% × item 2 + 15% × item 3 + 45% × item 4. (**D**) Mortality of mice infected via i.c. injection. The statistical significance of survival was analyzed using a survival curve and the log-rank (Mantel–Cox) test. (**E**) Weight changes in mice infected via i.p. injection. (**F**) Clinical symptom scores of mice infected via i.p. injection. (**G**) Mortality of mice infected via i.p. injection; all three data sets for virus-infected groups are nudged 1–3 units in the Y direction (with zero shift in the X direction) to make all traces for each group visible on the graph.

As outlined in Fig. 1A, 3-week-old Kunming mice were inoculated intracerebrally (i.c.) or intraperitoneally (i.p.) with TMUV strains CQW1, CHN-YC, or SCS01. For both injection routes, mice in the DMEM-treated (i.e., mock) group did not show any clinical symptoms and death. For i.c. injections, all affected mice exhibited similar clinical signs, including depression, ruffled fur, loss of appetite, reduced movement, hind limb paralysis, and difficulty keeping their eyes open due to excessive discharge. However, strain-dependent virulence patterns were observed according to the scores of clinical symptoms and survival rate. As shown in Fig. 1B through D, the CHN-YC virus demonstrated maximal neurovirulence with rapid disease progression: significant weight loss began at 4 days post-infection, with 100% mortality (6/8 dead at 6 dpi and 2/8 dead at 7 dpi) and the highest clinical score (=5.75). In contrast to CHN-YC, the CQW1 strain exhibited intermediate virulence with delayed weight loss onset, reduced mortality (37.5%, 3/8), and moderate clinical scores (=2.85). The SCS01 strain showed attenuated pathogenicity, inducing minimal yet statistically significant weight loss (*P* < 0.05 vs. mock) with 12.5% mortality (1/8 at 8 dpi, *P* > 0.05 vs. mock) and low clinical score (=0.9). Notably, i.p. inoculation with any strain failed to induce measurable clinical symptoms or mortality (Fig. 1E through G). Overall, the CQW1 strain exhibited stronger pathogenicity in mice compared with SCS01 but weaker than CHN-YC.

**Fig 2:**
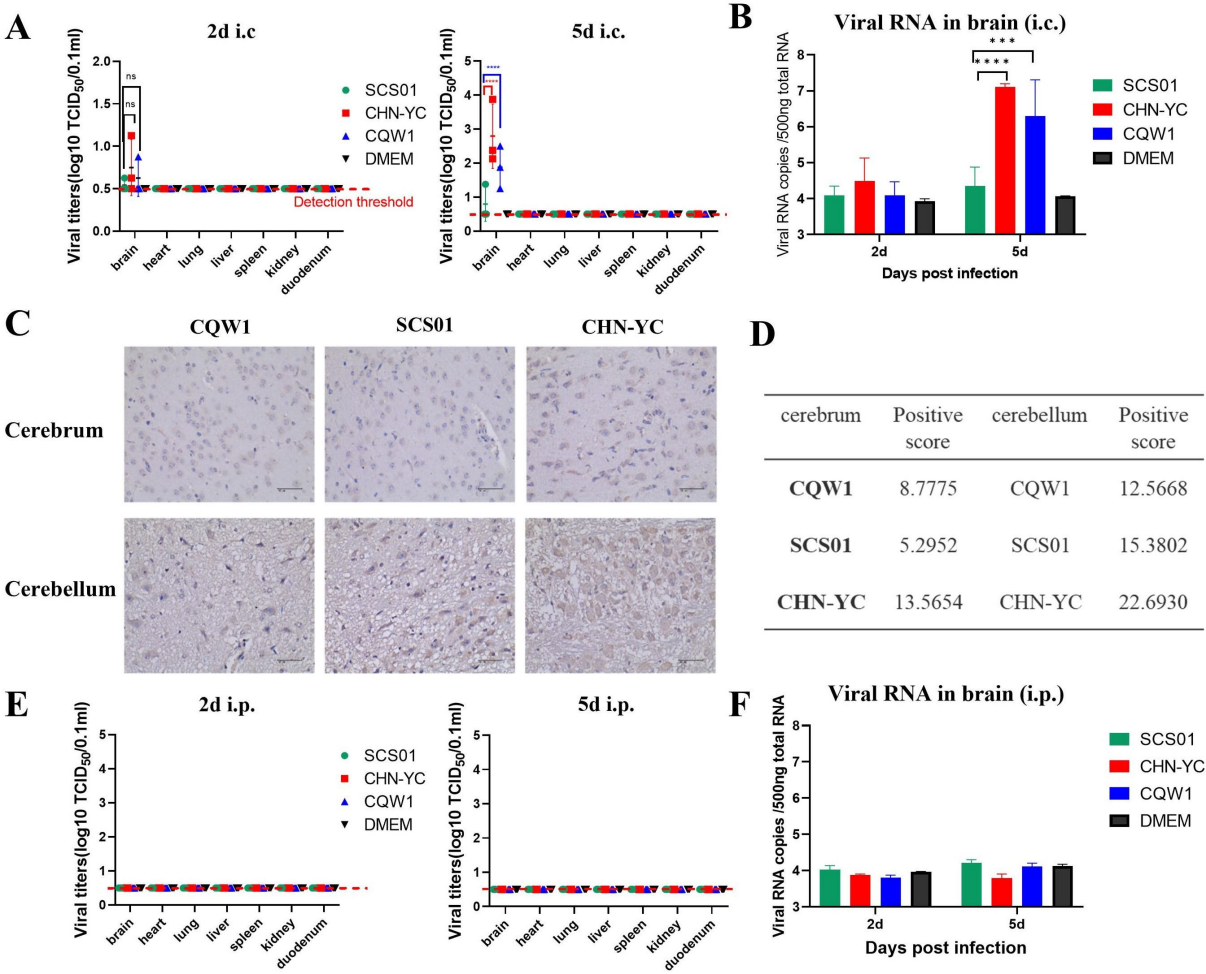
Viral characteristics of CHN-YC, SCS01, and CQW1 *in vivo*. (**A**) Viral loads in the brain, heart, lung, liver, spleen, kidney, and duodenum of mice at 2 and 5 days post-infection via the i.c. route. The red dotted line indicates threshold detection (=0.5). (**B**) Viral copy number in mice brains via the i.c. route, detected by RT-qPCR. (**C**) Immunohistochemical analysis at 5 days post-infection. (**D**) Immunohistochemistry scores corresponding to panel (**C**). (**E**) Viral loads in the brain, heart, lung, liver, spleen, kidney, and duodenum of mice at 2 and 5 days post-infection via the i.p. route. (**F**) Viral copy number in mice brains via the i.p. route. Means and SDs are shown. One-way ANOVA was performed to evaluate the statistical significance of viral titer.

To monitor viral replication *in vivo*, viral loads in different tissues were assessed using the TCID_50_ method (*n* = 3) at 2 and 5 dpi (Fig. 2A). Except for the brain, the initial injection site, no virus was detected in the heart, lung, liver, spleen, kidney, or duodenum after infection with any of the three viruses. This indicates that TMUV does not cause systemic infection via i.c. injection. SCS01 consistently showed a low infection rate of 1/3. CQW1 exhibited significantly higher titers than SCS01, whereas CHN-YC showed the most robust replication, reaching a high titer of 10^3.875^ for one of the mice at 5 dpi. Viral loads in the brain were further confirmed by RT-qPCR (Fig. 2B) and immunohistochemistry (Fig. 2C and D), revealing similar results. Notably, no virus was isolated from any of the sampled tissues after i.p. injection (Fig. 2E and F) using the TCID_50_ method, indicating that TMUV could not establish effective infection via the i.p. route. Additionally, neither RT-qPCR nor TCID_50_ detected virus in the brains of i.p.-infected mice.

Overall, these results suggest that different TMUV strains exhibit varying levels of neurovirulence in mice.

### Histological changes and immune and inflammation response post-TMUV infection

**Fig 3:**
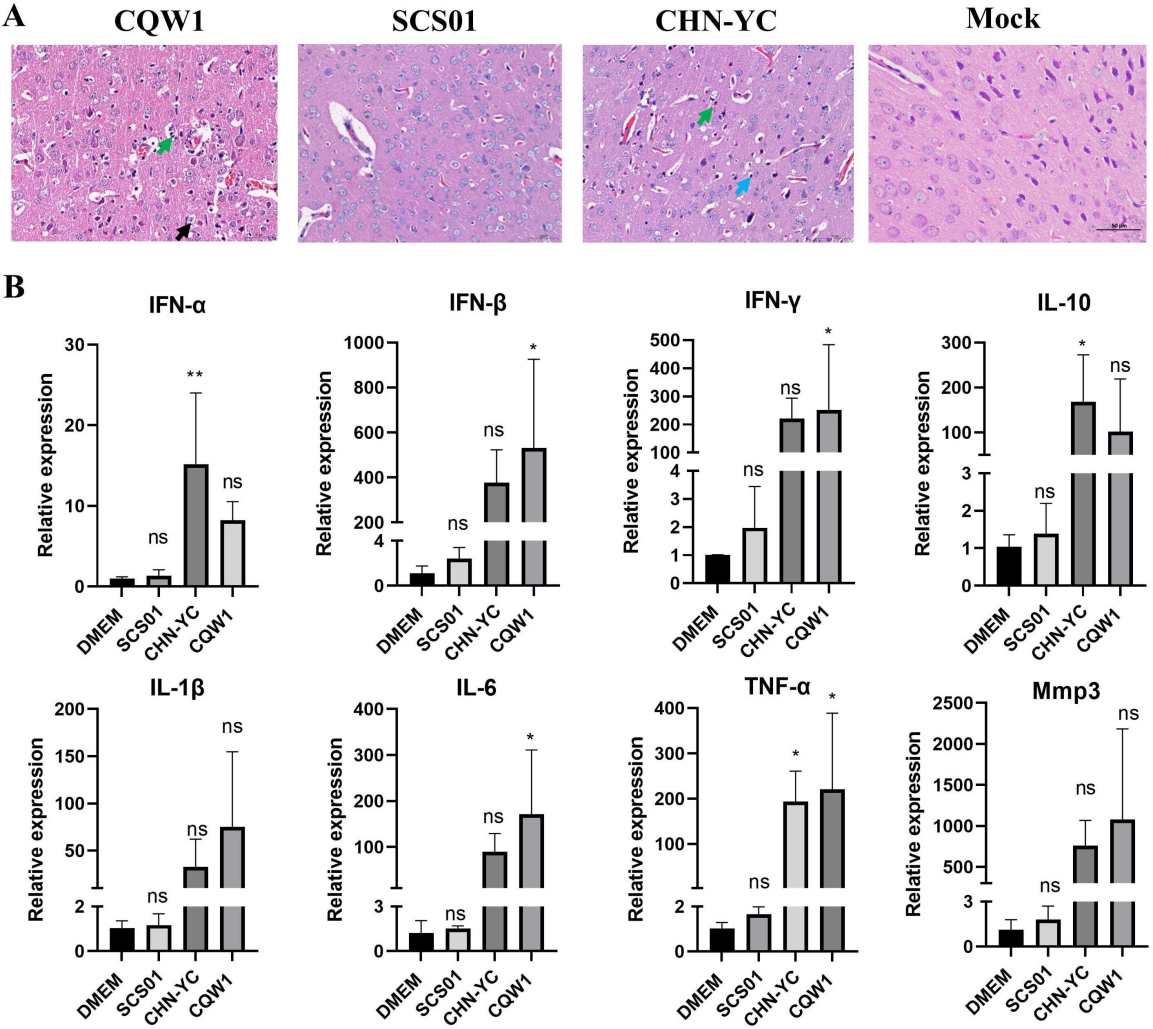
Histological changes and immune and inflammation responses post-TMUV infection. (**A**) Histopathological analysis of the cerebrum in mice infected with CHN-YC, SCS01, or CQW1. Neuronal degeneration and necrosis (green arrow), gliocyte proliferation (blue arrow), and neutrophils (black arrow) are indicated. (**B**) mRNA levels of IFN-α, IFN-β, IFN-γ, IL-1β, IL-6, TNF-α, IL-10, and Mmp3 in the cerebrum were measured by RT-qPCR. Means and SDs are shown; one-way ANOVA test was performed to evaluate the statistical significance of mRNA expression.

At 7 days post-infection, histological examination was performed to analyze the brain tissue from diseased mice. All three viruses caused similar pathological changes, displaying characteristics of encephalitis such as neuron degeneration, necrosis, gliocyte proliferation, and inflammatory cell infiltration (Fig. 3A).

Considering that neuronal injury is closely correlated with brain inflammation, we next analyzed the mRNA levels of cytokines and inflammatory factors post-TMUV infection (Fig. 3B). These included interferons (IFN-α, IFN-β, and IFN-γ), proinflammatory factors (IL-1β, IL-6, and TNF-α), the anti-inflammatory cytokine (IL-10), and matrix metalloproteinases 3 (which contribute to blood-brain barrier disruption) after TMUV infection. As shown in Fig. 3B, the upregulation of related genes was consistent with the severity of clinical symptoms and virus loads in the brain. Nearly all detected genes were significantly upregulated after infection with CHN-YC or CQW1 compared with the mock-treated group. In contrast, no significant differences were detected in the SCS01 virus-infected group. It should be noted that only one out of three mice was successfully infected in the SCS01 group, with a low virus titer (as shown in Fig. 2A); for another two mice, it seems inoculated SCS01 viruses did not overcome the mouse’s innate immunity and successfully established infection. These results indicated that the more virulent strain induced a higher level of cytokine and inflammatory factor expression.

### *In vitro* properties of the three TMUVs

**Fig 4:**
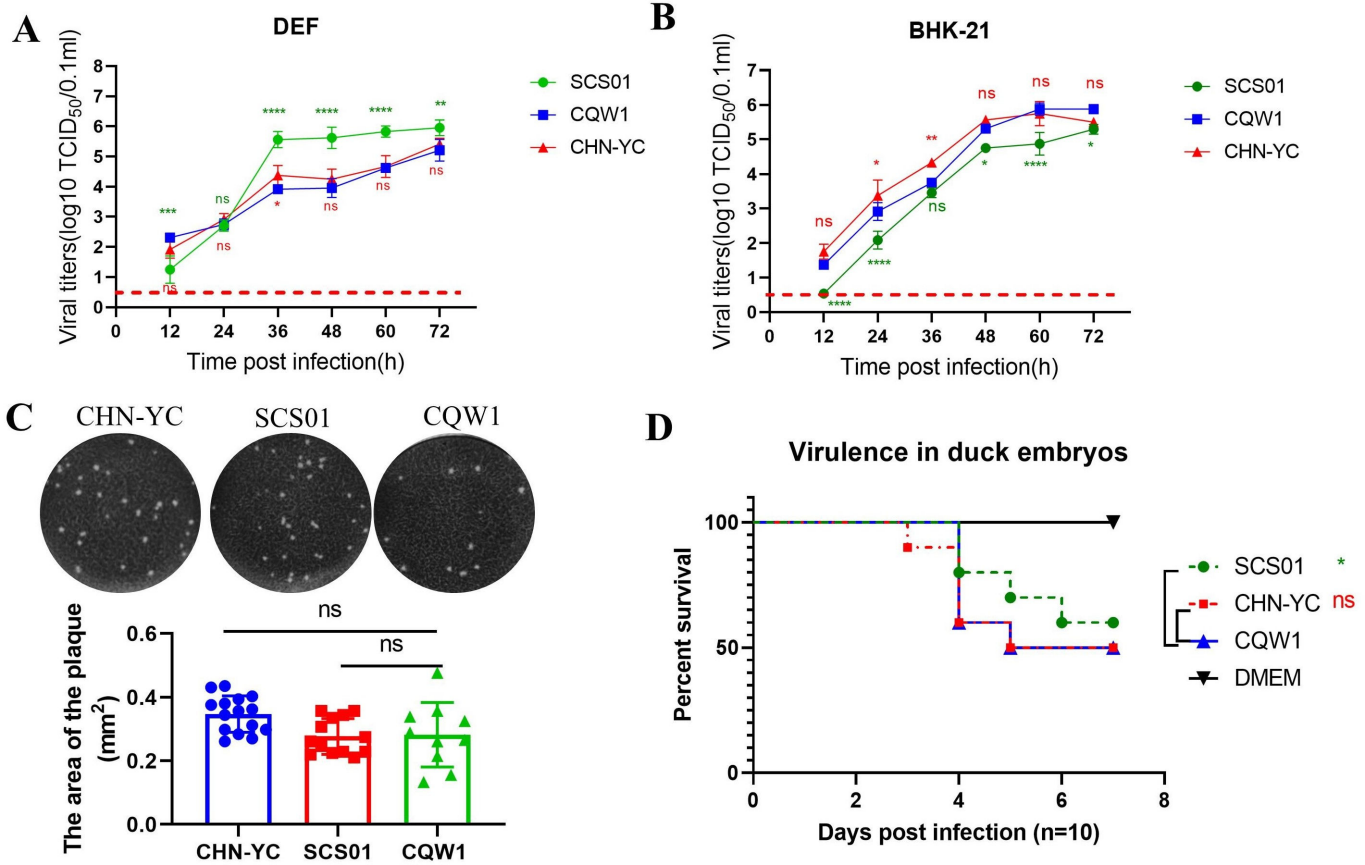
*In vitro* properties of the three TMUV strains. (**A**) Viral growth curves of CHN-YC, SCS01, and CQW1 in BHK-21 cells. (**B**) Viral growth curves in DEF cells. (**C**) Plaque morphology on BHK-21 cells. (**D**) Virulence of TMUVs in 9-day-old duck embryos. Each embryo was infected with 100 µL of CHN-YC, SCS01, or CQW1 at a dose of 10^3^ TCID_50_/0.1 mL (*n* = 10). Means and SDs are shown; a one-way ANOVA test was performed to evaluate the statistical significance and the statistical significance of plaque size; a two-way ANOVA test was performed to evaluate the statistical significance of the viral growth curve.

We next assessed the *in vitro* properties of the three TMUVs. All three viruses replicated efficiently *in vitro* (Fig. 4A and B). CHN-YC showed growth kinetics comparable with CQW1 on both DEF and BHK-21 cells. SCS01 displayed the most robust replication on DEF cells but showed the lowest titers on BHK-21 cells. However, there was no significant difference in the size of plaque among the three viruses (Fig. 4C). We further assessed their virulence in duck embryos. The CQW1 and CHN-YC strains showed similar virulence, whereas SCS01 displayed slightly attenuated virulence (Fig. 4D). These results indicate that different strains of duck TMUV indeed possess distinct characteristics *in vitro*.

### Genetic variations among different TMUV strains

**Fig 5:**
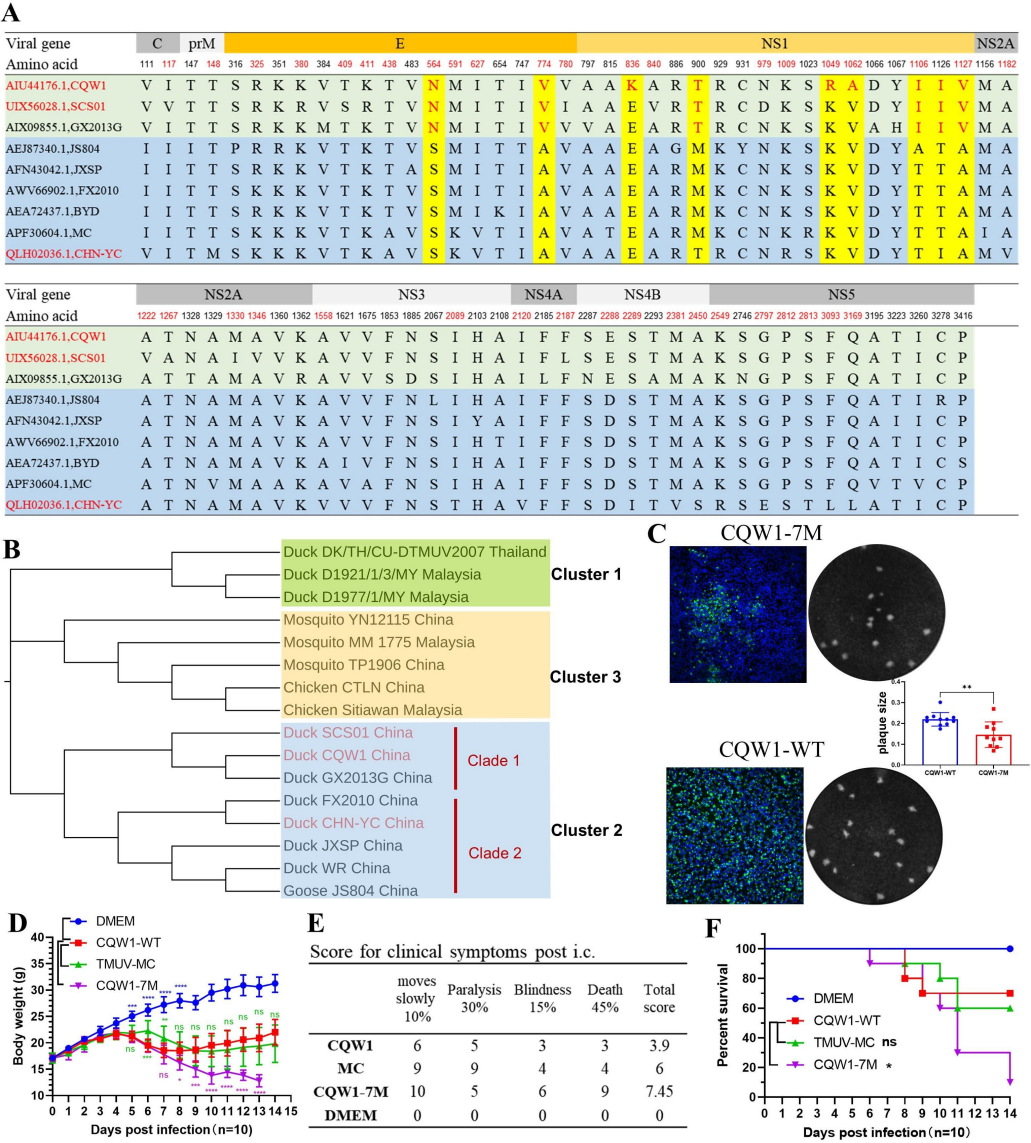
Evolutionary mutations enhance CQW1’s pathogenicity in mice. (**A**) Multiple sequence alignment (using Geneious 4.8 software) of 9 TMUV strains showing that all the unique mutations of CQW1 and SCS01 are located on the ORF of the E-NS1 region. (**B**) Phylogenetic tree analysis based on E protein sequences of TMUV, generated using the neighbor-joining method (Geneious 4.8 software). (**C**) Plaque morphology of CQW1-7M on BHK-21 cells. (**D**) Weight changes in Kunming mice post-infection with CQW1-7M (at a dose of 10^4.5^ TCID_50_/0.1 mL, *n* = 10). (**E**) Clinical symptom scores of infected mice. (**F**) Mortality of infected mice via intracranial injection. Means and SDs are shown. Student’s t test was used to evaluate the statistical significance of plaque size.

To investigate the viral determinants for the altered pathogenicity in mice, we aligned the polyprotein sequences of the CQW1, CHN-YC, and SCS01 strains. SCS01 showed high sequence similarity with CQW1, with only 15 amino acid differences recorded (Fig. 5A). A comparison of the three polyproteins revealed a total of 39 amino acid variations distributed throughout the entire viral polyprotein (Fig. 5A, highlighted in red font). Notably, 19 amino acid differences were located in the E-NS1 region of the polyprotein, accounting for nearly 50% (19/39) of all variations.

To gain more genetic information and narrow the screening range, we retrieved and aligned the sequences of another four TMUV early strains (Fig. 5A), which have been reported to be highly virulent to mice by i.c.. All of these strains used for multiple alignment analysis are isolated in ducks and have no recorded passage in mouse brain. Compared with these early strains, nearly all unique substitutions of CQW1 and SCS01 were located in the E-NS1 region of the ORF (Fig. 5A, highlighted in yellow). We identified nine naturally occurring amino acid substitutions in these early strains, including two amino acids in the E protein (E-N277S, E-V487A) and seven amino acids in the NS1 protein (NS1-K48E, NS1-T112M, NS1-R261K, NS1-A274V, NS1-I318T, NS1-I338T, and NS1-V339A). Further phylogenetic analysis indicated that CQW1 shared a clade with SCS01, belonging to Cluster 2.1, whereas CHN-YC and the other four early strains (JXSP, JS804, BYD, and FX2010) were grouped into Cluster 2.2 (Fig. 5B). Among the nine substitutions, E-N277 and NS1-I318 are specific to Cluster 2.1; E-V487 along with NS1-I338 is also conserved in Cluster 2.1; NS1-K48, NS1-R261 and NS1-A274 are specific to the CQW1 strain; NS1-M112 is specific to Cluster 2.2; and NS1-V339 seems to have no evolutionary specificity.

Considering the role of the E and NS1 proteins in flavivirus replication and pathogenesis, we focused on these substitutions in the subsequent sections.

### CQW1-7M greatly enhances pathogenicity in mice

Using reverse genetics technology, seven mutations (excluding NS1-V339A, which has no evolutionary specificity, and NS1-R261K, the latter is the same type of basic amino acid substitution) were engineered into the viral genome of CQW1, generating a chimeric virus designated as CQW1-7M. Upon transfection into BHK-21 cells, viral protein expression was detected by IFA, with a fluorescent signal observed at 3 days post-transfection. However, CQW1-7M formed smaller sizes of plaques compared with those produced by wild-type CQW1 (CQW1-WT) on BHK-21 cells (Fig. 5C), indicating that the CQW1-7M virus was successfully rescued.

Next, we evaluated the pathogenic potential of the recombinant CQW1-7M virus, comparing it with CQW1-WT and the naturally occurring Cluster 2.2 strain, MC virus, which endogenously possesses these seven mutations. Infected mice exhibited distinct disease severity (Fig. 5D through F). CQW1-7M caused the most significant weight loss and clinical severity (clinical score = 7.45), with a mortality rate of 90% (9/10, *P* < 0.05 vs. CQW1-WT). The MC strain showed intermediate virulence, resulting in 40% mortality (4/10, ns vs. CQW1-WT) and moderate clinical scores (score = 6). CQW1-WT exhibited attenuated pathogenicity, with lower weight loss, minimal clinical symptoms (score = 3.9), and 30% mortality (3/10). Despite differences in severity, all infected groups developed similar neurological symptoms, including anorexia, lethargy, blepharitis, and hindlimb paralysis (Fig. 5E). These results suggest that the seven mutations within the E-NS1 gene are responsible for the enhanced neurovirulence observed in mice.

### E-V487A increased the viral neurovirulence of CQW1 in mice

**Fig 6:**
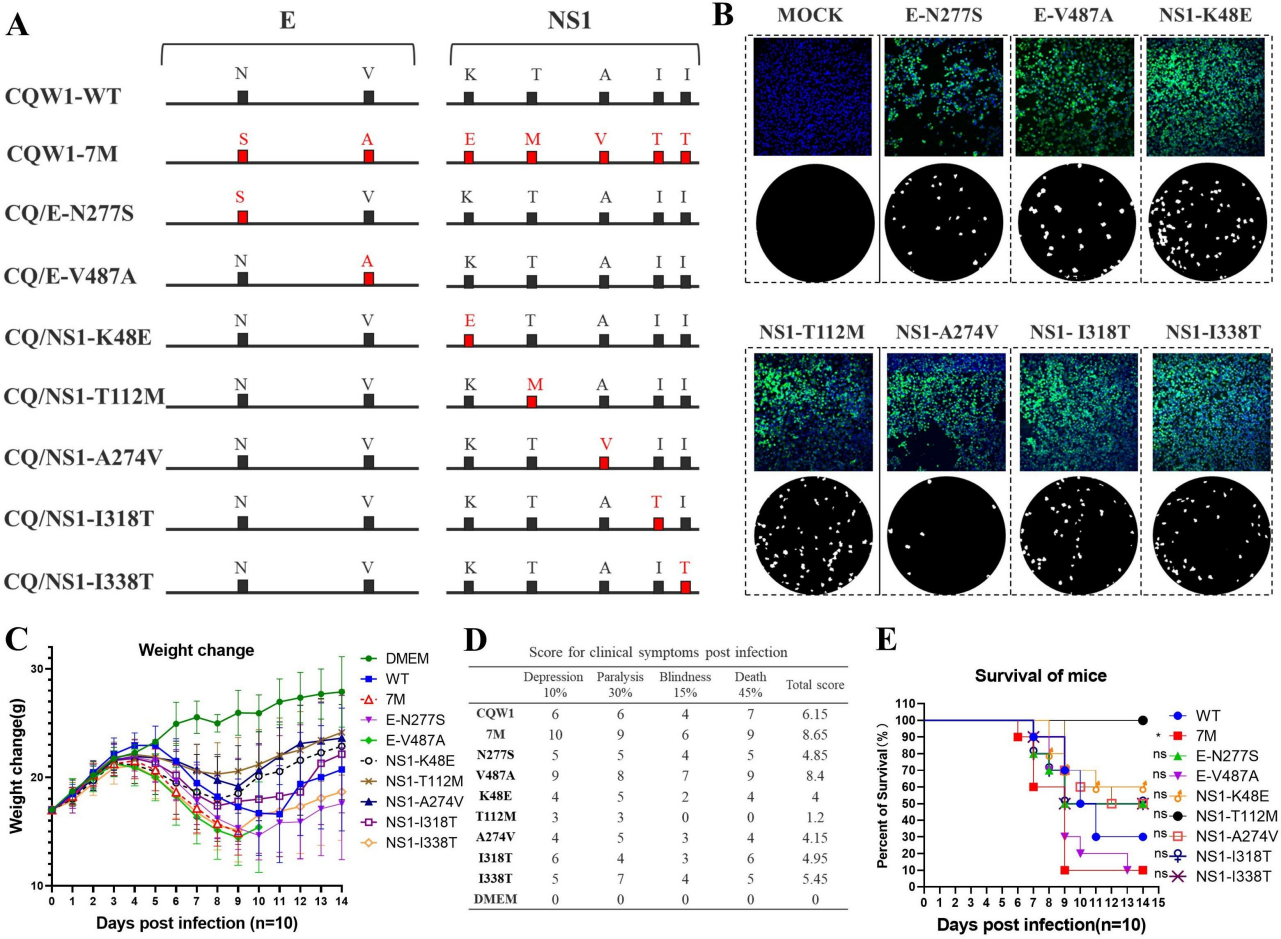
The E-V487A substitution increased the viral neurovirulence of CQW1 in mice. (**A**) Schematic diagram for recombinant TMUV with a single mutation. (**B**) Recovery of recombinant single-mutation viruses confirmed by IFA and plaque assay. (**C**) Weight changes in Kunming mice post-infection with CQ/E-N277S, CQ/E-V487A, CQ/NS1-K48E, CQ/NS1-T112M, CQ/NS1-A274V, CQ/NS1-I318T, and CQ/NS1-I338T (at a dose of 10^5.5^ TCID_50_/0.1 mL, *n* = 10). (**D**) Clinical symptom scores of mice infected with single-mutation viruses. (**E**) Mortality of mice infected with single-mutation viruses. Means and SDs are shown.

To determine the key amino acid that contributes to the changed neurovirulence of TMUV in mice, infectious cDNA clones for seven single-mutation viruses (CQ/E-N277S, CQ/E-V487A, CQ/NS1-K48E, CQ/NS1-T112M, CQ/NS1-A274V, CQ/NS1-I318T, and CQ/NS1-I338T) were generated (Fig. 6A). Upon transfection into BHK-21 cells, all seven single-mutation viruses were successfully recovered, as indicated by IFA results and plaque assays (Fig. 6B).

We further analyzed the neurovirulence of these single-mutation viruses in mice. All infected mice displayed a series of clinical symptoms similar to those previously described. Monitoring weight changes revealed that only the infections with CQW1-E-N277S or CQW1-E-V487A caused weight reductions as severe as those caused by CQW1-7M, particularly the latter (Fig. 6C). Correspondingly, the mice in the CQW1-V487A group exhibited the most severe symptoms, with the highest clinical symptom score compared with the other six single-mutation viruses (Fig. 6D). At 14 days post-infection, only 10% (1/10) of mice survived CQW1-V487A infection, matching the survival rate of CQW1-7M (Fig. 6E). All other six single-mutation viruses showed attenuated neurovirulence compared with CQW1-WT, as indicated by the clinical symptom scores and survival rates of the mice. Interestingly, the NS1-T112M substitution significantly attenuated the neurovirulence of CQW1, causing only mild clinical symptoms and no mortality. Altogether, these data indicate that the E-V487A substitution is responsible for the increased neurovirulence in mice.

### E-V487A increased virus replication *in vivo* and *in vitro*

**Fig 7:**
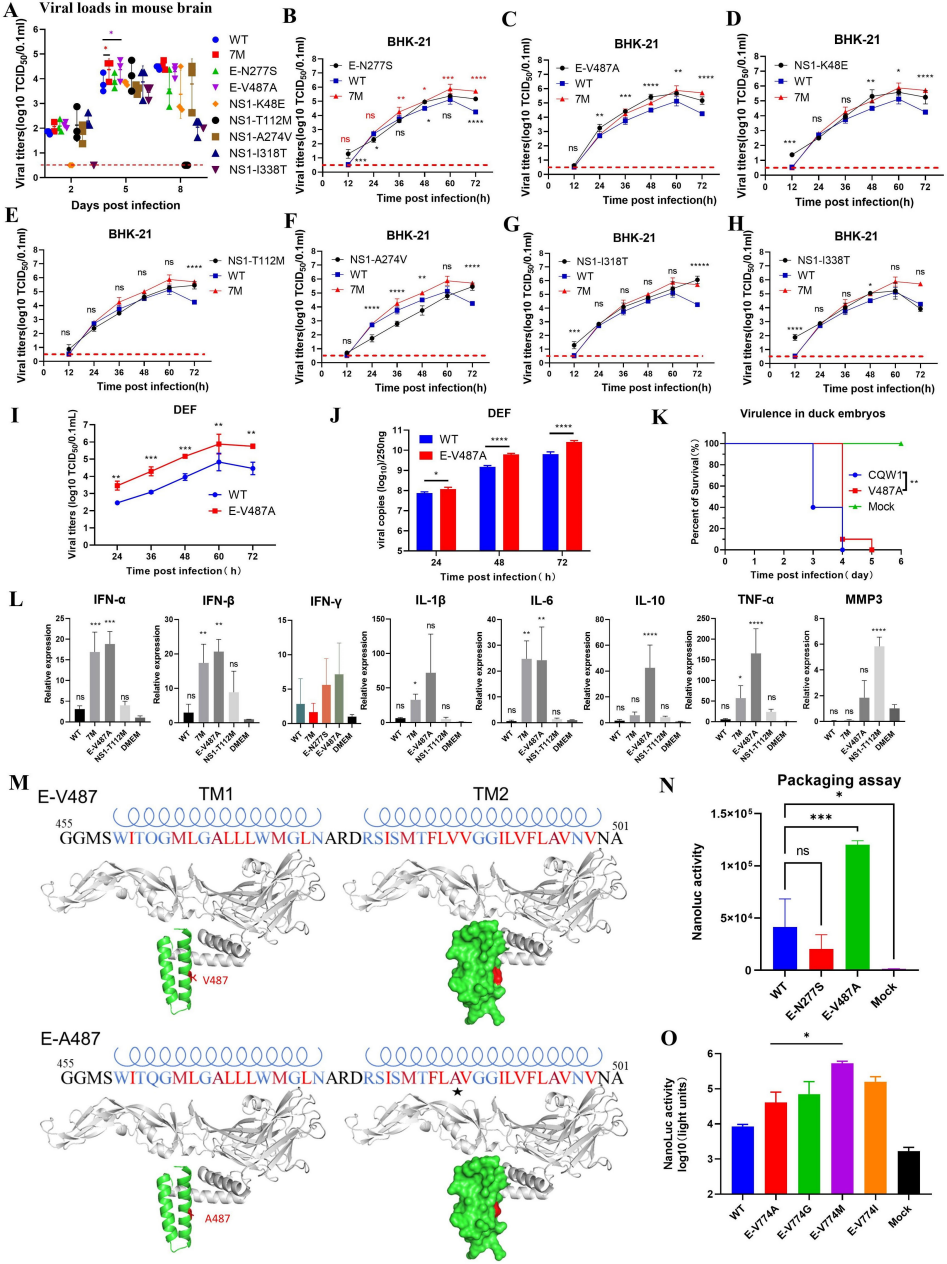
E-V487A mutation increased virus replication *in vivo* and *in vitro*. (**A**) Viral loads in mouse brains at 2, 5, and 8 days post-infection. (**B-H**) Viral growth curves of CQ/E-N277S, CQ/E-V487A, CQ/NS1-K48E, CQ/NS1-T112M, CQ/NS1-A274V, CQ/NS1-I318T, and CQ/NS1-I338T in BHK-21 cells. (**I**) Viral growth curves of CQ/E-V487A on DEF cells. (**J**) Viral RNA copies of CQ/E-V487A on DEF cells. (**K**) Virulence of CQ/E-V487A in 9-day-old duck embryos. Each embryo was infected with 100 µL virus at a dose of 10^3^ TCID_50_/0.1 mL (*n* = 10). (**L**) mRNA levels of IFN-α, IFN-β, IFN-γ, IL-1β, IL-6, TNF-α, IL-10, Mmp2, and Mmp3 in the cerebrum measured by RT-qPCR. (**M**) Homology model of the CQW1 E protein generated using SWISS-MODEL, based on the West Nile virus E protein structure (7kv9). Hydrophobic amino acids are indicated in red. (**N**) Replicon packaging assay assessing the effect of E-V487A on viral assembly and release, and viral assembly efficiency was measured by NanoLuc activity. (**O**) The effect of the 487^th^ amino acid of E protein on viral assembly. Means and SDs are shown. A one-way ANOVA test was performed to evaluate the statistical significance of packaging assays.

We next determined the viral kinetics of these single-mutation viruses *in vitro* and *in vivo*. All viruses replicated effectively in mouse brains, but CQW1-7M and CQ/E-V487A produced relatively higher titers than all other viruses at 5 days post-infection, displaying the most robust replication kinetics (Fig. 7A). Interestingly, the CQ/NS1-T112M virus was quickly cleared at 8 days post-infection, much faster than all others. Further analysis of their growth kinetics in BHK-21 cells showed that CQW1-7M replicated more robustly than the CQW1-WT virus (Fig. 7B through H). All single-mutation viruses replicated effectively in BHK-21 cells. Among them, CQ/E-V487A displayed slightly enhanced replication compared with CQW1-7M. CQ/E-N277S and CQ/NS1-K48E showed comparable growth kinetics with CQW1-7M, whereas CQ/NS1-T112M, -I318T, and -I338T showed comparable growth kinetics with CQW1-WT. Only CQ/NS1-A274V attenuated virus replication. Furthermore, the V487A mutation enhanced viral proliferation in DEF cells (Fig. 7I and J) but slightly reduced virulence in duck embryos (Fig. 7K). Collectively, these data confirm that the E-V487A substitution significantly enhances CQW1 proliferation both *in vivo* and *in vitro*.

The immune and inflammatory responses were also determined at 5 days post-infection (Fig. 7L). Generally, CQW1-7M and CQ/E-V487A induced higher levels of innate immune and pro-inflammatory cytokines than CQW1-WT or CQ/NS1-T112M. The first two viruses caused higher mortality and more severe clinical signs. These data again highlight the relevance of immune and inflammatory responses to the outcome of TMUV infection via i.c. injection.

The 487^th^ amino acid residue is located in the middle of the transmembrane domain II (TM2) of the Tembusu virus E protein, a region rich in hydrophobic amino acids (Fig. 7M), which is a common feature among different flaviviruses. Considering that TM2 has been proven to play a role in the viral assembly process, and the V487A substitution enhanced the proliferation of the CQW1 virus *in vitro* and *in vivo*, we examined the impact of V487A on viral assembly using a replicon packaging assay (Fig. 7N). As shown, compared with the WT construct, V487A significantly increased the packaging efficiency of prM/E, whereas the E-N277S substitution had no effect on assembly. Considering the hydrophobic character of the transmembrane helix, the V487A substitution may change the local hydrophobicity of TM2, influencing viral packaging efficiency. To further confirm the role of the 487^th^ amino acid residue in viral assembly, we engineered additional mutations using the replicon packaging assay. Interestingly, all three mutations significantly increased viral assembly efficiency, especially the substitution with Met, which generated the highest NanoLuc activities (Fig. 7O). These data indicate that the 487^th^ amino acid plays an essential role in the viral assembly process.

## DISCUSSION

TMUV is an emerging avian flavivirus causing severe egg-drop syndrome and encephalitis in avian species, particularly ducks and chickens (5, 19, 20). Its zoonotic potential is worthy of attention, but the information on neurovirulence and pathogenesis of TMUV in avian species and mammalian hosts remains relatively limited.

TMUV exhibits age-dependent neuroinvasiveness in suckling mice but lacks pathogenicity in 3-week-old and older mice when administered intraperitoneally (9). The reasons behind this discrepancy may be complex: our data (Fig. 1) and a previous study (7) both demonstrated that TMUV cannot establish an effective systemic infection in the peripheral organs of mice via i.p. infection. Although TMUV replicates effectively in the brains of mice after i.c. inoculation, no live virions were detected in other organs using the TCID_50_ detection method. Similar results have been reported using plaque detection (7), where only viral RNA was found in peripheral organs, again indicating that TMUV is unable to establish an effective systemic infection. Consequently, limited infection in peripheral organs may not support the virus invasion of the CNS. Considering that TMUV is highly sensitive to mammalian interferon (9), as the innate immune system matures with age, mice develop stronger resistance to TMUV infection, resulting in age-dependent neuroinvasiveness. Additionally, the blood-brain barrier also fully develops with age, which may play an important role in preventing virus invasion into the brain, although this point needs more experimental data to confirm.

Our earlier work showed that the prototypical mosquito-derived TMUV, MM_1775 strain, is highly virulent to mice via the i.c. inoculation, causing 100% mortality (23). Similar neurovirulence was observed for several duck TMUVs isolated in 2010, which belong to Cluster 2.2 (7, 9). However, in the present study, our data indicated that the neurovirulence of Cluster 2.1 strains is significantly attenuated compared with Cluster 2.2 TMUV, especially the recent isolate SCS01 strain, which only caused mild symptoms and did not result in any mouse mortality. The severity of the disease also correlated tightly with the replication ability in the mouse brain. Among the three strains, the SCS01 strain showed the lowest replication level and only induced mild upregulation of cytokines. In contrast, the CHN-YC strain exhibited the most robust replication level and induced much higher levels of cytokines, suggesting a correlation between disease severity and cytokine levels. An excessive amount of cytokines may affect the brain, leading to neural symptoms.

Nearly all human-pathogenic flaviviruses are zoonotic. For TMUV, it maintains a life cycle in poultry (ducks, geese, and chickens) or arthropods, but accidental spillover infection in humans may not be uncommon. A study demonstrated a surprisingly high rate of seroconversion and positive viral RNA in duck farm workers in China (11). A high rate of neutralizing antibody response to TMUV was also observed in people residing around farming areas and in the general population in Thailand (12). In recent years, the genetic diversity of TMUV circulating in China has increased, and its host range has expanded. A recent report confirmed that the goose-origin HQ-22 strain (belonging to Cluster 3) could cause severe neurological symptoms and death in ICR mice via intranasal infection (22). The increased infectivity of TMUV to mice via intranasal infection, suggesting the potential risk of TMUV infection in mammals (and even in humans), should warrant our attention.

In the present study, we confirmed that a single amino acid substitution in the transmembrane domain (TMD) of the E protein is responsible for the neurovirulence variation between duck TMUV genotype Clusters 2.1 and 2.2. Although both Cluster 2.1 and Cluster 2.2 viruses emerged from a common ancestor, different environmental pressures shaped their distinct genomic characteristics. Multiple sequence alignment and phylogenetic analysis highlighted that all common variations of Cluster 2.1 are located on the E-NS1 gene compared to Cluster 2.2. To date, no data systematically analyze the *in vivo* and *in vitro* characteristics of different genotypes of duck TMUVs, although viruses from both clusters are primarily isolated from diseased ducks.

In our study, we demonstrated that Cluster 2.1 TMUV showed attenuated neurovirulence in mice compared with Cluster 2.2 TMUV, with a single amino acid substitution E-V487A being responsible for this changed phenotype. The E protein is the major antigen of flaviviruses and plays an essential role in viral attachment, entry, assembly, and budding processes, thereby determining viral cell tropism and pathogenesis. The transmembrane domain of the flavivirus E protein is an α-helical hairpin, with the 487^th^ amino acid located on its TM2. It has been demonstrated that interactions of the double anchor of the flavivirus E protein contribute substantially to particle assembly, stability, and maturation (30). Hence, the polarity change caused by the E-V487A substitution could influence the intra-TMD interactions, which are required for subsequent steps contributing to capsid integration for virion formation. Additionally, due to the hydrophobic interior of the lipid bilayer, the amino acid residues in TMDs are often hydrophobic. The E-V487A substitution changes the local hydrophobicity of the TMD, which may affect its insertion into the membrane and stability, ultimately impacting virion assembly.

Altogether, we demonstrated that a single amino acid substitution located in the transmembrane domain of the E protein is responsible for the altered neurovirulence of different genotypes of duck TMUV. These findings will help in understanding the molecular mechanisms of TMUV pathogenesis in avian and mammalian hosts.

## Data Availability

All data to understand and assess the conclusions of this study are available in the published article. The raw data that support the findings of this study are available from the corresponding author upon reasonable request.
